# Isolation of the Autoinducer-Quenching Strain that Inhibits LasR in *Pseudomonas aeruginosa*

**DOI:** 10.3390/ijms15046328

**Published:** 2014-04-14

**Authors:** Lixing Weng, Yuqian Zhang, Yuxiang Yang, Lianhui Wang

**Affiliations:** 1 School of Geography and Biological Information, Nanjing University of Posts and Telecommunications, Nanjing 210046, Jiangsu, China; 2 Jiangsu Key Laboratory for Organic Electronics & Information Displays and Institute of Advanced Materials (IAM), Nanjing University of Posts and Telecommunications, 9 Wenyuan Road, Nanjing 210046, Jiangsu, China; E-Mail: 082023005@fudan.edu.cn; 3 Department of Microbiology and Microbial Engineering, School of Life Sciences, Fudan University, Shanghai 200433, China; E-Mail: 09210700014@fudan.edu.cn

**Keywords:** quorum sensing, *Pseudomonas aeruginosa*, *Agrobacterium tumefaciens* NT1, biofilm, virulence factor

## Abstract

Quorum sensing (QS) has been recognized as a general phenomenon in microorganisms and plays an important role in many pathogenic bacteria. In this report, we used the *Agrobacterium tumefaciens* biosensor strain NT1 to rapidly screen for autoinducer-quenching inhibitors from bacteria. After initial screening 5389 isolates obtained from land and beach soil, 53 putative positive strains were identified. A confirmatory bioassay was carried out after concentrating the putative positive culture supernatant, and 22 strains were confirmed to have anti-LasR activity. Finally, we determined the strain JM2, which could completely inhibit biofilm formation of *Pseudomonas aeruginosa* PAO1, belonged to the genus *Pseudomonas* by analysis of 16S rDNA. Partially purified inhibitor factor(s) F5 derived from culture supernatants specifically inhibited LasR-controlled elastase and protease in wild type *P. aeruginosa* PAO1 by 68% and 73%, respectively, without significantly affecting growth; the *rhl*-controlled pyocyanin and rhamnolipids were inhibited by 54% and 52% in the presence of 100 μg/mL of F5. The swarming motility and biofilm of PAO1 were also inhibited by F5. Real time RT-PCR on samples from 100 μg/mL F5-treated *P. aeruginosa* showed downregulation of autoinducer synthase (LasRI and *rhlI*) and cognate receptor (*lasR* and *rhlR*) genes by 50%, 28%, 48%, and 29%, respectively. These results provide compelling evidence that the F5 inhibitor(s) interferes with the *las* system and significantly inhibits biofilm formation.

## 1. Introduction

*Pseudomonas aeruginosa* is one of the most difficult pathogens to treat clinically, and infects vulnerable patients including those with postoperative immune suppression. In patients with cystic fibrosis (CF), *P. aeruginosa* causes lung disease or death. This pathogen exhibits intrinsic resistance to many structurally unrelated antibiotics [1]. Quorum sensing (QS) is a population density-dependent regulatory system that regulates the secretion of pathogenic virulence factors and biofilm formation in *P. aeruginosa*. It is thought that interfering with QS will inhibit microbial pathogenicity [2,3].

In *P. aeruginosa*, there are three interconnected QS systems: the *las*, *rhl*, and *pqs* systems [4–6]. The major signal molecules involved in these three QS systems are 3OC12-homoserine lactone, C4-homoserine lactone, and 2-heptyl-3-hydroxy-4-quinolone (PQS), respectively [6,7]. Among them, the *las* QS system is at the top of the QS hierarchy, and regulates the *rhl* and *pqs* QS systems [8]. *N*-(3-oxododecanoyl)-l-homoserine lactone (3OC12-HSL, OdDHL) is produced by the LasI AHL synthase in the *las* system. Once OdDHL reaches a critical threshold concentration, it binds to transcriptional regulatory protein LasR. Dimers of OdDHL-LasR then bind to target promoters and upregulate the expression of downstream genes such as protease and elastase genes. The *rhl* system consists of *N*-butanoyl-l-homoserine lactone (C4-HSL, BHL), the cognate receptor RhlR, and the synthase RhlI. Virulence factors such as pyocyanin and rhamnolipid are mainly regulated by the *rhl* system. The *las* and *rhl* systems control a complicated regulatory network involving several hundred genes [9].

Infections of *P. aeruginosa* are of great concern because of its increasing resistance towards conventional antibiotics. QS in *P. aeruginosa* acts as a global regulator of almost all virulence factors, including biofilm formation [10]. As the QS system of *P. aeruginosa* directly relates to its pathogenesis, targeting the QS systems will provide an improved strategy to combat drug resistance in this organism. Small molecule chemicals called quorum sensing inhibitors (QSIs) can selectively act on the receptors at the cell surface of bacteria, or directly penetrate the cell membrane to interact with the enzymes or proteins of various signal transduction cascades, eventually interfering with pathogenicity. Recently, there have been reports of QSIs specific for *Pseudomonas aeruginosa*. It was reported that bromohalogenated furanones from marine red alga *Delisea pulchra* effectively suppressed *P. aeruginosa* biofilm formation by interfering with QS [1]. Patulin and penicillic acid from *Penicillium* spp can enhance *P. aeruginosa* biofilm sensitivity to tobramycin, and activate neutrophilic granulocytes to remove the bacteria in a mouse model of *P. aeruginosa* infection [11]. A variety of bioactive agents, both natural and synthetic, were recently reported to have significant anti-biofilm activity against Gram positive and negative bacteria [12,13]. One synthesized QSI molecule, *N*-decanoyl-l-homoserine benzyl ester, was found to down-regulate total protease and elastase activities, as well as the production of rhamnolipid, without affecting bacterial growth. It had synergistic interactions with several antibiotics, including tobramycin, gentamycin, cefepime, and meropenem [14].

In previous reports, we constructed a rapid plate method to screen for QSIs from bacteria, using two biosensor strains, *Agrobacterium tumefaciens* NT1 for OdDHL inhibitors and *Chromobacterium violaceum* CV026 for BHL inhibitors [15,16]. The purple pigment violacein in *Chromobacterium violaceum* CV026 (Kmr cviI::mini-Tn5) is inducible by AHL with *N*-acyl side chains from C4 to C8 in length. An isolate identified as a *Pseudomonas* sp. was capable of inhibiting violacein production according to the *C. violaceum* CV026 bioassay. A more highly purified preparation (4 μg/mL) from concentrated culture supernatants of this isolate specifically inhibited *rhl*-controlled pyocyanin and rhamnolipid production in wild type *P. aeruginosa* PAO1 by 49%, without significantly affecting growth. The inhibitor reduced protease activity by about 46% but had no effect on biofilm in PAO1 [17]. QS is a key mechanism that regulates several aspect of biofilm development, including adhesion, motility, maturation, and dispersal [18–20]. In searching for novel quorum-quenching bacteria from soil samples, we focused on screening the *las* QS system, and obtained an isolate that strongly inactivated autoinducing activity and reduced the PAO1 biofilm formation. The compound produced by this isolate could potentially be a biological control for biofilm infection. *A. tumefaciens* NT1 (traR, tra::lacZ749) displays the broadest sensitivity to AHLs at the lowest concentrations, and senses AHL with *N*-acyl side chains in long length from 6 to 12 carbons as well as three unsubstituted AHLs [21,22]; we used *A. tumefaciens* NT1 as the reporter strain for *las* system inhibitor isolation in this study. A description of a novel autoinducer-quenching strain is presented here, including the anti-LasR fragment from culture supernatant extract, and its inhibition of biofilm formation and QS dependent virulence factors. We also describe its phylogenetic position based on 16S rRNA gene sequence information. At present, a therapy that efficiently targets bacterial biofilm does not exist, since biofilms are inherently resistant to conventional antibiotics. The threat of resistance development with these drug candidates is uncommon, as they attenuate only the virulence factors and not the growth of the pathogen [8,10,14]. In the present study, we targeted the *las* system of *P. aeruginosa* and studied its inhibition upon exposure to bioactives from one bacterium (JM2). This study also emphasizes the potential of JM2 to produce bioactive agents with anti-LasR and anti-biofilm properties that are novel drug candidates.

## 2. Results and Discussion

### 2.1. Isolation of the Anti-LasR Strain

#### 2.1.1. Detection of Anti-LasR on Solid Medium

**Figure 1.**
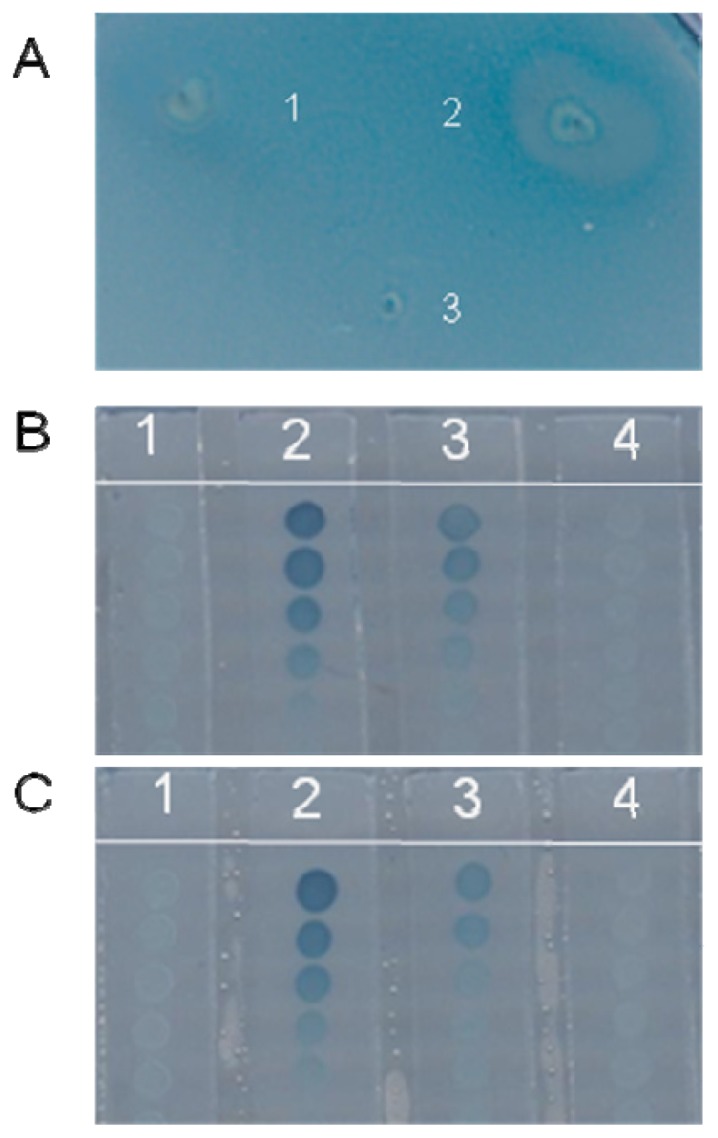
Isolation of bacteria expressing anti-LasR QS system activity. (**A**) Agar plate screening assay; the diffuse and lightly colored areas surrounding the strain indicate inhibition of OdDHL production. Strain 2 caused a strong degree of inhibition, but did not affect the growth of NT1. Strains 1 and 3 showed no inhibitory activity; (**B**) Inhibition of OdDHL production in a secondary screen. 1: methanol as a negative control; 2: OdDHL 100 nM; 3: OdDHL 100 nM plus extract from JM2, the extract from JM2 showed strong inhibitory activity; 4: extract from JM2 showed no autoinducer activity; (**C**) Inhibition of OdDHL production for the JM2 partially purified extract F5. 1: methanol as a negative control; 2: OdDHL 100 nM; 3: OdDHL 100 nM and F5, F5 showed stronger inhibitory activity than the crude extract; 4: extract from JM2 showed no autoinducer activity.

For bacterial screening, the test isolates from soil were first inoculated using sterile toothpicks onto their appropriate media and incubated overnight at 28 °C [16]. In the initial plate screening after incubation overnight, a blue color in the indicator bacteria NT1 occurred without inhibitors. Many test isolates grew well and had a blue-colored background, which indicated that there was no particular compound inhibiting the action of exogenously added OdDHL. In some instances, a small, cloudy, and colorless circle appeared around the test bacteria, indicating that *las* system repressors were being produced by the test strain. Figure 1 shows the colorless circle of bacteria on the NT1 plate (Figure 1A) designated JM2. Among more than 5000 bacteria isolates from land and beach, we obtained 53 isolates that showed a very obvious, colorless, opaque halo in the NT1 assay. Being cloudy indicated the growth of the reporter stain was not affected, and being colorless indicated that the production of β-galactosidase in NT1 was not successfully induced, therefore indicating that the QS signaling had been disturbed. Several possible reasons existed for how the QS signaling was disrupted: (1) the generation of QS autoinducer signals was inhibited; (2) the QS signals were metabolized or degraded; or (3) the signal molecules were prevented from activating the LasR transcription. The latter possibility was the focus of this study; we chose to explore whether the signal inhibitor chemicals produced by the test bacteria functioned in this capacity. We chose to study test bacteria with a colorless, but viable circular zone produced on the lawn of NT1, because these test strains had no effect on the growth of the reporter strain, which indicated no antibiotic products were being secreted by the test isolates. Growth inhibition would produce a clear halo *versus* a cloudy halo, while QS inhibitors will permit growth but inhibit only the hydrolysis of X-gal, showing a colorless, non-transparent zone around the target strain.

#### 2.1.2. Further Bioassay of the Putative Strains

Out of more than 5000 bacterial strains screened preliminarily for anti-LasR activity, 53 strains showed different levels of QS inhibition. We isolated these 53 putative anti*-*LasR strains for supernatant extraction and evaluation with further bioassays. Twenty-two isolate extracts demonstrated anti-LasR activity. One strain extract had stronger inhibition than all other isolates; this isolate was purified further on YEB agar plates by streaking, and the purified species was designed as JM2.

As shown in Figure 1B, there was no colored colony at the blank agar slice. The length of colored colonies at the agar slice of extract of JM2 and 10 μM OdDHL was shorter than that of 10 μM OdDHL alone, demonstrating strong inhibition of QS. The length of blue colonies is inversely proportional to the autoinducer-quenching activity. At the same time, we found that methanol had no effect on QS, and the JM2 extract had no effect on reporter strain growth (data not shown). Therefore, we determined that the active compounds produced by JM2 were not antibiotics. The negative effect on blue pigment production on the bioassay plate was not caused by inhibition of growth, but by disruption of QS signaling systems (Figure 1B).

### 2.2. Bioassay Guided Fractionation of JM2 Methanol Extract

Following the screening of fractionation effectiveness, compounds were tested in a concentration dependent manner. Figure 1B shows the results of these experiments in which varying concentrations of JM2 methanol extract were used. The anti-LasR component activity was more pronounced in a concentration gradient of bioactive fraction F5 (0.2–1.0 mg/mL). Fraction F5 showed anti-QS activity even at 10 μg/mL, as shown by a 72% (*p* < 0.001) reduction in blue pigment production by the NT1 reporter strain (Figure 1C).

### 2.3. Construction of lasR Deletion Mutant (lastR^−^)

The deletion vector was first introduced into DH5α and subsequently into S17-1 by transformation because DH5α has a high transformation efficiency, while S17-1 has a high conjugation efficiency. Transconjugants that contain the deletion vector in the PAO1 chromosome were recovered from the gentamicin-containing MM agar, and streaked onto MM agar containing 10% sucrose. Sucrose is detrimental to the suicide vector due to the SacB region, thus, causing the vector region to be excised from the chromosome. The sucrose-resistant colonies were then screened using colony PCR to identify the deletion mutants. The resulting positive PCR results were subsequently reconfirmed by repeating PCR on the possible mutants. Sequencing of the possible mutant was carried out to confirm the lasR^−^ mutant when the control samples failed.

### 2.4. Effect of F5 on the Production of Virulence Factors in P. aeruginosa PAO1

#### 2.4.1. Effect of F5 on Pyocyanin, Elastase, Rhamnolipids and Protease Production in *P. aeruginosa* PAO1 and LasR^−^ Mutant

**Figure 2.**
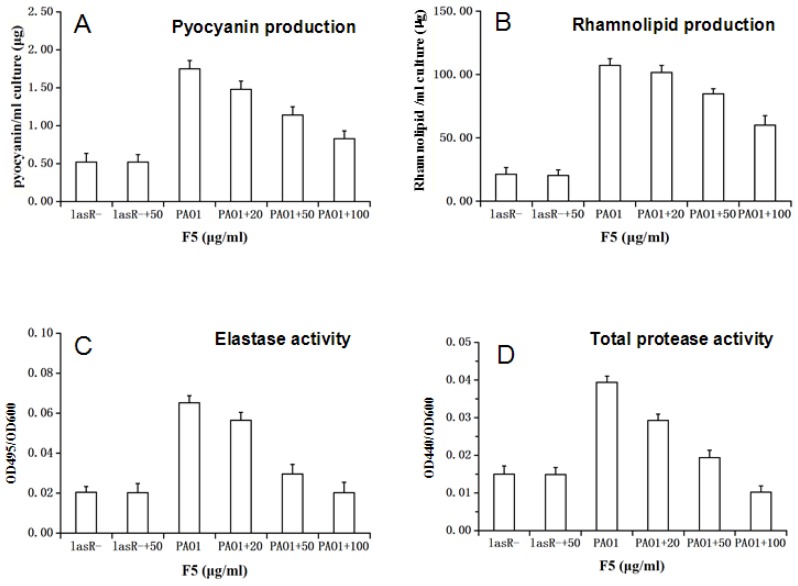
The effect of partially pure inhibitor F5 on the virulence factors in PAO1 and *lasR*^−^ mutant. Effect of serial dilutions of the final extract on pyocyanin production (**A**); rhamnolipid production (**B**); elastase activity (**C**); and protease activity (**D**) in PAO1.

*P. aeruginosa* virulence factors were reduced by the bioactive fraction F5 in PAO1; significant reduction (*p* < 0.001) in pyocyanin (54%), elastase (68%), rhamnolipids (52%), and protease (73%) production in the presence of 100 μg/mL of F5 was observed. In lasR^−^ mutant, these virulence factors dropped strongly and even added F5 (Figure 2). All assays were done in triplicate, and the values were expressed as mean ± SD.

#### 2.4.2. Effect of F5 on Biofilm Formation

**Figure 3.**
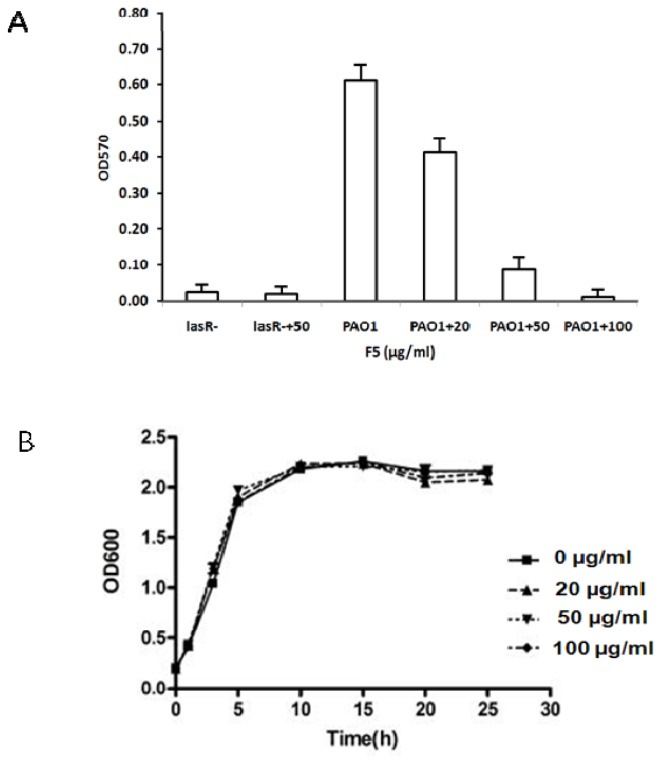
Bioassay testing effect of F5 on biofilm formation and growth. (**A**) Measurements of effects of F5 serial dilutions on biofilm formation in PAO1 and *lasR^−^* mutant. Blank control was set by addition of methanol, F5 at 0 μg/mL as CK (control), 20, 50, and 100 μg/mL, respectively; (**B**) Effect of F5 on planktonic growth of *P. aeruginosa* PAO1. Cells were grown in LB medium, in the presence of different concentrations of F5 at 0 μg/mL as control, 20, 50, 100 μg/mL, respectively. The data represent mean values of three independent experiments.

The PAO1 strain was able to develop biofilms on a plastic surface, with OD_570_ values ranging from 0.6 to 0.67, but *lasR*^−^ mutant was not (Figure 3A). The effects of F5 on *P. aeruginosa* biofilm formation were further evaluated using a crystal violet-based biomass-staining assay. At the concentration of 100 μg/mL, F5 caused a 95% decrease in the ability of PAO1 to form biofilms, relative to PAO1 grown without F5 (Figure 3A). With lower concentrations (20 μg/mL) of F5 there was a slight inhibitory effect on biofilm formation (Figure 3A); F5 at 50 μg/mL caused a larger decrease in biofilm formation (82% decrease) (Figure 3B). All assays were done in triplicate, and the values were expressed as mean ± SD (Dunnett’s test, *p* < 0.01). We performed a growth curve analysis for *P. aeruginosa* PAO1 cells exposed to 100 μg/mL of F5, and these cells showed no lag in growth as compared with control cultures containing only media and the solvent (methanol) used for compound resuspension (Figure 3B). These data suggest that F5 affects biofilm formation independent of cell growth and propagation.

#### 2.4.3. Effect of F5 on Swarming Assay

**Figure 4.**
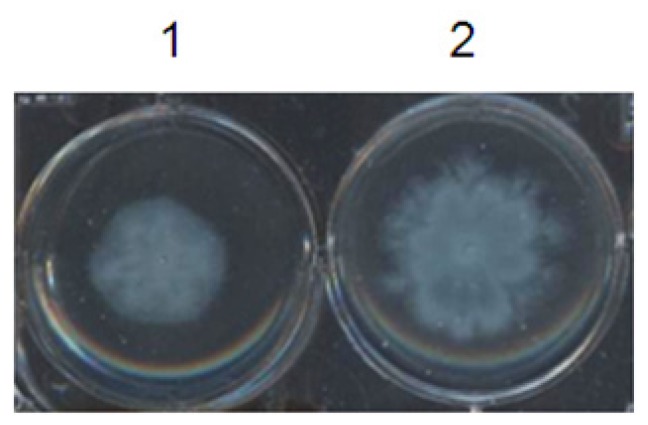
Effect of F5 on swarming assay of *P. aeruginosa* PAO1. Swarming agar plates supplemented with 50 μg/mL F5 (**1**), without F5 (**2**) were stab inoculated with a sterile needle to the bottom of the medium and incubated for 24 h at 37 °C.

Microorganisms with motility are able to move to find more favorable or less hazardous niches for colonizing and persisting in a given environment. Swarming is a special kind of motility observed on semi-solid surfaces, defined as surface translocation dependent on extensive flagellar motion and cell-to-cell connections. It has an important role in the avoidance of harmful environments, and in colony formation. Swarming motility in *P. aeruginosa* requires QS because it uses rhamnolipids that act as a biosurfactant. Rhamnolipid production is dependent on and regulated by QS systems. The results shown in Figure 4 indicate that F5 is capable of inhibiting swarming motility in PAO1 cells grown in medium. The observed difference in colony diameters (Figure 4) shows the effect of F5 on the *P. aeruginosa* QS system. When F5 was added to the culture medium, no effect on microbial growth was observed at the assayed concentrations. Swarming was limited at these concentrations, but not fully inhibited. Figure 4 shows images of swarming cells taken after 24 h of culture. PAO1 colonies have edges with fan-shaped or finger-shaped protrusions, and, under our experimental conditions, colony diameters of 2.2 to 2.5 cm. After culturing the cells with the addition of 50 μg/mL F5 for 24 h, the swarming ability of PAO1 was significantly weakened. The colony edges, as compared with the control group, appeared smooth and nearly circular, and the colony size shrunk to a diameter of 0.8 to 1.1 cm.

### 2.5. Phylogenetic Analysis

**Figure 5.**
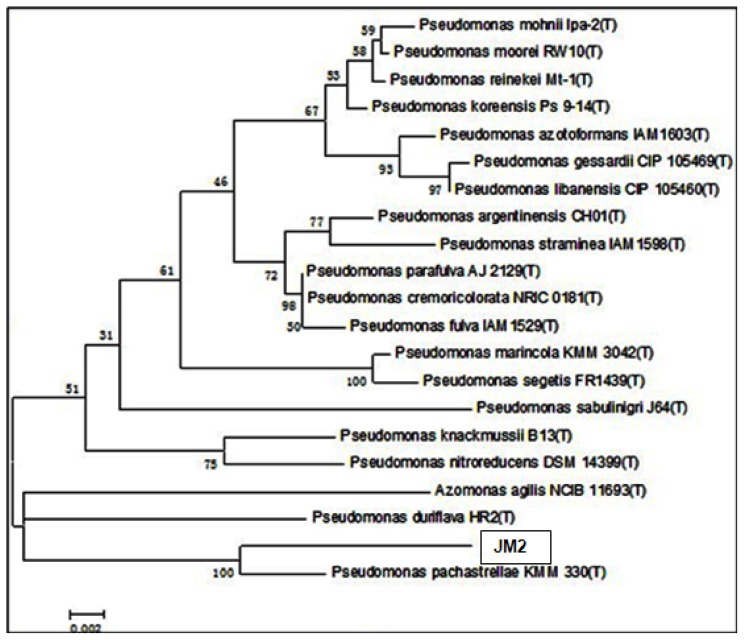
Phylogeny of strain JM2. Phylogenetic maximum-parsimony tree calculated using partial 16S rDNA gene sequences. Maximum-parsimony (1000 resamplings) bootstrap values are provided for relevant groups. The bar indicates 0.002% sequence divergence. The numbers provide support for the robustness of the adjacent nodes.

To establish the phylogenetic position of the isolate JM2, the 16S rDNA gene from JM2 was sequenced. The accession number in NCBI BankIt is ID 1666536 ACCESSION BSeq#1. The JM2 16S rDNA sequences were aligned with published GenBank 16S rDNA sequences, and homologous comparison results showed that the JM2 16S rDNA sequence shared 98.8333% homology with 16S rDNA from the marine bacterium *Pseudomonas pachastrellae* (Figure 5), and was more than 95% identical to 16S rDNA from bacteria in the *Pseudomonas* genus. Therefore, JM2 is most closely related to *Pseudomonas pachastrellae* KMM330 (T).

### 2.6. Real Time RT-PCR

**Figure 6.**
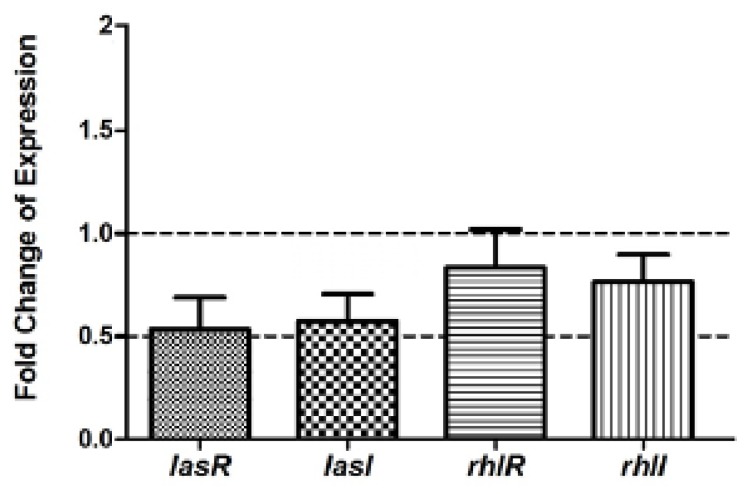
Comparison of *lasR*, *lasI*, *rhlR*, *rhlI* expression levels with F5 treatment relative to expression of the internal control gene *rpsL* in real time RT-PCR.

Real time RT-PCR showed a 50%, 48%, 21%, and 25% reduction in the expression of *lasI*, *lasR*, *rhlI* and *rhlR* genes, respectively, upon treatment with 100 μg/mL of F5 (Figure 6). Our results reveal a new class of bacterial biofilm inhibitor, and further support an approach to biofilm inhibition via inhibition of the *las* QS system. Although much success in drug discovery has been reported using chemical molecules from plants, the likelihood of finding a novel drug from this collection of molecules is extremely low. This prompted us to embark on a study using microbes with various bioactive potentials. Studies are underway to explore the specific mechanism of action of the F5 compound(s) in biofilm inhibition, and to further identify the molecular structure(s) and activity.

We found that the isolated strain JM2, which was belonged to the genus Pseudomonas, can efficiently interfere with the quorum-sensing signaling circuit in wild type *P. Aeruginosa* PAO1. A partially purified the inhibitor factor(s) F5 derived from the culture supernatants specifically inhibited *las*-controlled biofilm, elastase and protease in PAO1. Real-time polymerase chain reaction analysis showed that F5 downregulated the transcriptions of autoinducer synthase (*lasI* and *rhlI*) and their cognate receptor (*lasR* and *rhlR*) genes, which resulted in attenuation of QS-regulated virulence activities, such as biofilm formation, and secretion of protease, elastase and pyocyanin. The protease and elastase are las-controlled virulence factors [23], pyocyanin and rhamnolipid is controlled by rhl system [24]. The reduction data of elastase (69%), protease production (73%), pyocyanin (53%), rhamnolipids (51%) showed that F5 have stronger effect on lasR than on rhlR. Further, the PAO1 las system contributes greatly to biofilm formation substantial down-regulated transcription of lasR and lasI may be directly responsible for the reduction of PAO1 biofilms [25,26]. These results suggest that F5 from JM2 may possess lasR inhibitory activity against the virulence of PAO1.

## 3. Experimental Section

### 3.1. Bacterial Strain and Medium

*Agrobacterium tumefaciens* NT1 was used as an indicator for OdDHL, contained a tra-lacZ fusion, and expressed β-galactosidase activity in the presence of a recognized autoinducer [22]. The medium for the NT1 bioassay was solid minimal medium (MM), comprised of (per 1 L) 10.5 g K_2_HPO_4_, 4.5 g KH_2_PO_4_, 0.2 g MgSO_4_·7H_2_O, 5 mg FeSO_4_, 10 mg CaCl_2_, 2 mg MnCl_2_, 2 g (NH_4_)_2_SO_4_, 2 g d-Mannitol, 15 g agar; MM was adjusted to pH 7.2. When necessary, a solution (5 mL) containing 1.5% (*w*/*v*) agar and 40 μg/mL 5-bromo-4-chloro-3-indoyl-β-d-galactopyranoside (X-gal) was overlaid onto the minimal medium plates. YEB agar medium contained (per 1 L) 5 g sucrose, 5 g yeast extract, 0.5 g MgSO_4_·7H_2_O, 10 g tryptone, 5 g NaCl, 15 g agar; YEB was pH adjusted to 7.0–7.2 with 0.1 M NaOH. R2A medium was composed of 0.5 g of yeast extract, 0.5 g of peptones (pancreatic digest of casein, 50%; and peptic digest of animal tissue, 50%), 0.5 g of acid hydrolysate of casein, 0.5 g of dextrose, 0.5 g of soluble starch, 0.3 g of K_2_HPO_4_, 0.05 g of MgSO_4_·7H_2_O, and 0.3 g of sodium pyruvate per 1 L (pH adjusted to 7.0 with K_2_HPO_4_ and KH_2_PO_4_); solidified with 0.8% (*w*/*v*) agarose when required. OdDHL was synthesized as described previously [27].

### 3.2. Isolation of Anti-LasR Bacterium

Bioassays for determining the anti-LasR strains were performed as described previously [14,16]. In initial color screening for anti-LasR activity, we employed the biosensor strain NT1 for long-chain AHL inhibitor detection. NT1 generates blue color by decompounding X-gal when induced to produce β-galactosidase by AHL. Soil samples collected from China [16], were suspended in sterilized water (50 mL) and spread over YEB agar plates in a ten-fold serial dilution. After incubation at 30 °C overnight, test colonies were selected randomly and inoculated into YEB solid medium and bioassay plates. Bioassay plates comprised of 0.1 × LB, 0.25 × LB, LB, or R2A media for different bacteria, were covered with an overlay of 5 mL of melted MM (50 °C) containing 30 μL of NT1 overnight culture, 20 nM OdDHL, and 300 μM X-gal. After the overlay solidified, test strains were inoculated onto the medium with a sterile toothpick. The plates containing X-gal required protection from light.

To prepare for purifying and identifying the anti-LasR molecules from the bacteria identified by primary screening, we further extracted culture supernatants. To ensure strain purity, isolates were streaked on YEB plates, and a single colony was used for further studies. The OdDHL inhibitor bioassay was performed on MM plates, and the extract from the test strain was used in the bioassay. Two milliliters overnight culture of test strain isolates were incubated in two liters of appropriate medium for appropriate time. Cultures were centrifuged and supernatant was collected, transferred to a new tube, and mixed 1:1 with acidified ethyl acetate. The mixture was shaken for 4 h. After quiescence and stratification, the aqueous and organic layers were separated. The organic layer was evaporated with a rotary evaporator, and the residue was dissolved in 400 μL of methanol. The concentrated extract was further screened using a slightly modified version of the previously described assay method [14]. Briefly, MM agar (15 mL) supplemented with 100 μM of X-gal was cut into separate slices (10 mm in width). A mixture (2 μL) of extract and synthetic autoinducer, after being incubated at 30 °C, was spotted on the top of the agar gel strips, and NT1 culture (OD_600_ ≅ 0.4) was inoculated on the remaining agar gel strips at an interval of 0.5 cm from the loaded samples using a sterilized tip. These solutions were spotted on the top of the slices as follows in the MM agar plate: 5 μL of extract and 5 of 10 μM OdDHL, 5 μL of extract and 5 μL of methanol as test samples; 10 μL of methanol as a blank control; 5 μL of methanol and 5 of 10 μM OdDHL as a negative control. The plates were protected from light and incubated at 37 °C overnight to elicit color change. The distance from the last induced blue colony to the origin of the loaded sample in each agar slice was measured. No inoculum was added to the mixed fluid sample at the top of agar gel strip to serve as a negative control. Blue colonies indicated the presence of autoinducer [22]. All bioassay experiments were performed in triplicate unless otherwise stated.

### 3.3. Extraction of Putative Anti-LasR Active Compound(s) from Spent Bacterial Culture Supernatants

Overnight JM2 culture (5 mL) was inoculated into 500 mL of LB broth. Cells were grown to an OD_600_ of 1.5, and cells were removed by centrifugation at 12,000× *g* for 15 min at 4 °C. The spent culture supernatant was extracted twice with an equal volume of ethyl acetate. The organic layer was collected in a separation funnel, evaporated with a rotary evaporator to dryness. Residues were dissolved in 100 μL of methanol and stored at −20 °C. Anti-LasR extracts were analyzed with biosensor NT1. The isolated strain was cultivated in 100 L LB medium batches, and 20 mL of extract in methanol was collected.

The isolated strain JM2 that exhibited strong anti-LasR activity was cultivated in 100 L LB medium batches and the culture supernatants were extracted as previously described [17]. Briefly, the extract was fractionated by passage through a silica column with three different sequential eluents (4:1 ethyl acetate/petroleum ether, ethyl acetate, methanol), yielding three active fractions as determined by the plate bioassay. Anti-LasR components were more concentrated in fraction 5 (designated as F5), as this fraction showed anti QS activity even at 20 μg/mL (data not shown). The successive F5 extracts were collected, combined, centrifuged, filtered, dried, and weighed (total of 15.8 mg collected).

### 3.4. Effect of F5 on Virulence Factors in P. aeruginosa PAO1

Virulence factor assays, including biofilm formation, swarming, total protease activity, elastase activity, pyocyanin, and rhamnolipid, were described in a previous study [14].

### 3.5. Effect of Pseudomonas aeruginosa Growth

Growth of PAO1 was monitored in the presence of different concentrations of F5 (0–1.0 mg/mL).

### 3.6. 16S rDNA Sequence Identified

JM2 DNA was extracted using a Takara kit, and primers 27F (5′-AGAGTTTGATCCTGGCTCAG-3′) and 1492R (5′-GGTTACCTTGTTACGACTT-3′) were used for the PCR amplification of 16S rDNA. The 16S rDNA sequences were analyzed using comparative analysis to GenBank sequences via the BLAST program, and the highest homology bacterial 16S rDNA sequences were selected for the genetic distance calculation, which was carried out using the PHYLIP program (Phylogeny Inference Package, Version 3.57c, Joseph Felsenstein, University of Washington, Seattle, WA, USA) to generate phylogenetic trees. To avoid misreading due to PCR errors, sequencing of the PCR fragment was repeated at least twice. The closely known relatives of the new JM2 isolate were determined.

### 3.7. Construction of lasR Deletion Mutant of PAO1

Deletion of *lasR* gene was carried out using an approach based on PCR. For the construction of deletion construct, two PCRs were performed, one amplifying the sequences upstream (f1: 5′-CCGGAATTCACGGTTTTCTTGAGCTGG-3′ and r1: 5′-GCGGGATCCAAGGCCAGTCC GGCACCG-3′) and the other amplifying the sequences downstream (f2: 5′-GCGGGATCC GAAAACCGGGCCGAGGCC-3′ and r2: 5′-GCTCAAGCTTGGCTTCACACGAGAGAAC-3′) of the deletion endpoints. For the upstream primers, df1 contains an *EcoR*I site, while dr1 contains a *BamH*I site. For the downstream primers, df2 contains a *BamH*I site, while dr2 contains a *Hind*III site. Amplification of the target gene region was carried out using the deletion primers in a PCR reaction mixture, which included 50 ng of PAO1 genomic DNA (template). Digestion was performed with *EcoR*I and *BamH*I, *Hind*III and *BamH*I, individually, PCR products were cloned into an *EcoR*I-*Hind*III-restricted pK18MobSacB-get vector, *via* a three-piece ligation procedure. DH5α Transformation was carried out using the heat shock method. The spread plate medium contained 50 μg per mL of gentamicin, 50 μg per mL of Xgal and 0.1 μM of IPTG. Individual white colonies were selected and subcultured. PCR was performed to identify successful DH5α transformants. PCR was carried out by amplification of the 1 kb deletion fragment contained in the suicide vector, using primers that were designed to anneal to the pK18MobSacB-get vector, at regions slightly upstream (forward primer 5′-AGTTTAAGAAGAACGTAG-3′) and downstream (reverse primer 5′-ACTTGTG CATCTCGCCCA-3′) of the deletion fragments endpoints. PCR conditions were: 5 min at 94 °C to denature the template, and then amplified by 30 cycles of 30 s at 94 °C, 45 s at 55 °C, and 45 s at 72 °C.

Positive plasmid DNA was extracted from DH5α transformants and introduced into *E. coli* S17-1 by transformation. Following S17-1 transformation, the deletion vector (pK18MobSacB-get suicide vector carrying the deletion fragment) was transferred into PAO1 via conjugation. The PAO1 cells were resuspended with 400 μL of MM. 70 μL of the resuspension were spread onto MM agar plates containing 300 μg of gentamicin. Colony PCR and sequencing were used to identify the deletion mutants. PCR conditions: 5 min at 94 °C to denature the template, followed by amplification by 30 cycles of 30 s at 94 °C, 45 s at 55 °C, and 3 min of extension time at 72 °C.

### 3.8. Mechanism of Anti-LasR Activity of Bioactive Fraction F5

To estimate the expression of QS genes, PAO1 was treated with bioactive fraction F5 and total RNA was extracted using TRIZOL (Sigma, St. Louis, MO, USA). First strand cDNA synthesis was done as described in the manufacturer’s protocol (Fermentas, Pittsburgh, PA, USA). Real time RT-PCR was done using SYBR green master mix. In 10 mL reaction mixture, 5 mL of SYBR green master mix, 100 ng of cDNA, 5 mM target gene primers (*lasI*, *lasR*, *rhlI* or *rhlR*) and 1 mM 16S rRNA primers (internal housekeeping gene) were used.

## 4. Conclusions

JM2 belonged to the genus *Pseudomonas* by analysis of 16S rDNA. The partially purified inhibitor factor(s) F5 from culture extraction could completely inhibit biofilm formation of *P. aeruginosa* PAO1, and inhibit other LasR-controlled virulence factors without significantly affecting growth. Real time RT-PCR result showed F5-treated *P. aeruginosa* PAO1 had downregulation of autoinducer synthase (LasRI and rhlI) and cognate receptor (lasR and rhlR) genes by 50%, 28%, 48%, and 29%, respectively. The evidence indicated that the F5 inhibitor(s) interfered with the *las* system and significantly inhibited the relative virulence.
